# Validation of a HLA-A2 tetramer flow cytometric method, IFNgamma real time RT-PCR, and IFNgamma ELISPOT for detection of immunologic response to gp100 and MelanA/MART-1 in melanoma patients

**DOI:** 10.1186/1479-5876-6-61

**Published:** 2008-10-22

**Authors:** Yuanxin Xu, Valerie Theobald, Crystal Sung, Kathleen DePalma, Laura Atwater, Keirsten Seiger, Michael A Perricone, Susan M Richards

**Affiliations:** 1 Genzyme Corporation, One Mountain Road, Framingham, Massachusetts, MA 01701, USA

## Abstract

**Background:**

HLA-A2 tetramer flow cytometry, IFNγ real time RT-PCR and IFNγ ELISPOT assays are commonly used as surrogate immunological endpoints for cancer immunotherapy. While these are often used as research assays to assess patient's immunologic response, assay validation is necessary to ensure reliable and reproducible results and enable more accurate data interpretation. Here we describe a rigorous validation approach for each of these assays prior to their use for clinical sample analysis.

**Methods:**

Standard operating procedures for each assay were established. HLA-A2 (A*0201) tetramer assay specific for gp100_209(210M) _and MART-1_26–35(27L)_, IFNγ real time RT-PCR and ELISPOT methods were validated using tumor infiltrating lymphocyte cell lines (TIL) isolated from HLA-A2 melanoma patients. TIL cells, specific for gp100 (TIL 1520) or MART-1 (TIL 1143 and TIL1235), were used alone or spiked into cryopreserved HLA-A2 PBMC from healthy subjects. TIL/PBMC were stimulated with peptides (gp100_209_, gp100_pool_, MART-1_27–35_, or influenza-M1 and negative control peptide HIV) to further assess assay performance characteristics for real time RT-PCR and ELISPOT methods. Validation parameters included specificity, accuracy, precision, linearity of dilution, limit of detection (LOD) and limit of quantification (LOQ). In addition, distribution was established in normal HLA-A2 PBMC samples. Reference ranges for assay controls were established.

**Results:**

The validation process demonstrated that the HLA-A2 tetramer, IFNγ real time RT-PCR, and IFNγ ELISPOT were highly specific for each antigen, with minimal cross-reactivity between gp100 and MelanA/MART-1. The assays were sensitive; detection could be achieved at as few as 1/4545–1/6667 cells by tetramer analysis, 1/50,000 cells by real time RT-PCR, and 1/10,000–1/20,000 by ELISPOT. The assays met criteria for precision with %CV < 20% (except ELISPOT using high PBMC numbers with %CV < 25%) although flow cytometric assays and cell based functional assays are known to have high assay variability. Most importantly, assays were demonstrated to be effective for their intended use. A positive IFNγ response (by RT-PCR and ELISPOT) to gp100 was demonstrated in PBMC from 3 melanoma patients. Another patient showed a positive MART-1 response measured by all 3 validated methods.

**Conclusion:**

Our results demonstrated the tetramer flow cytometry assay, IFNγ real-time RT-PCR, and INFγ ELISPOT met validation criteria. Validation approaches provide a guide for others in the field to validate these and other similar assays for assessment of patient T cell response. These methods can be applied not only to cancer vaccines but to other therapeutic proteins as part of immunogenicity and safety analyses.

## Background

Cancer immunotherapy clinical trials often use immunological assessment as secondary endpoints to evaluate vaccine potency. A number of techniques have been established to monitor antigen specific immunologic responses in patients. Many of these assays monitor T cell responses and were comprehensively reviewed by Keilholz et al. [1]. Most commonly used methods include: (1) direct measurement of serological cytokines, (2) T cell functional analysis for cell proliferative response, CTL, and cell associated cytokine production by Flow Cytometry and ELISPOT, and cytokine gene expression by real time RT-PCR, (3) cell phenotypic analysis (multi-color Flow Cytometry) including antigen specific T cell detection using HLA tetramers and additional cell phenotypic analysis for activated T cells, regulatory T cells (Treg), and naïve/memory T cells. Assay development studies (IFNγ Real Time RT-PCR and ELISPOT, HLA-A2 Tetramer analysis) and monitoring specific vaccine response in cancer patients are described by a number of investigators [2-10]. Although many different assays are used to monitor immune response in cancer patients, few of these assays are validated when used for clinical applications [1,3,11,12]. Furthermore, the validation of immunoassays was identified as one of the critical areas for improvement when using these assays to evaluate immune responses in the clinic [1].

Unlike assays used for research studies, clinical assays need to be simple and robust, with reasonable turn around time, and high throughput. Minimal sample manipulation during sample collection, processing, shipment, storage, and testing are added advantages. Assays requiring small sample volume are also preferable. Methods that meet these criteria are optimized for each component and step during assay development/pre-validation studies. Standard Operating Procedures (SOP) and assay validation plans with acceptance criteria are followed in validation studies to further assess assay performance characteristics. Regulatory agencies and published white papers provide guidance on validation of analytical methods and immunogenicity methods to monitor anti-protein drug antibody response. Less information is available for validation of flow cytometry and T cell functional assays, which are generally more challenging.

We developed and validated HLA-A2 flow cytometry, IFNγ real time RT-PCR, and IFNγ ELISPOT assays to monitor specific CD8^+ ^T cell responses in HLA-A2 melanoma patients immunized with genetic vaccines encoding glycoprotein 100 (gp100) or MART-1, two melanoma-associated antigens. We report our study on validation of the three methods using TIL cells alone or spiked into normal PBMC samples. The performances of the assays were further confirmed using PBMC from immunized patients. Assay performance met validation criteria and all three assays were shown to be effective for their intended use, monitoring patient's antigen specific T cell response.

## Methods

### TIL cells, Jurkat cells, and frozen PBMCs from healthy subjects and melanoma patients

#### TIL cells

Frozen CD8^+ ^TIL cells (isolated from HLA-A2 melanoma patients) were generously provided by Dr. Steven A. Rosenberg (NCI, NIH, Bethesda, MD) including TIL1520 (gp100 specific), TIL1235 (MART-1 specific), and TIL1143 (MART-1 specific). Each TIL cell line was expanded to generate a working cell bank. Cells were stored at -120°C in single use aliquots. Freshly thawed cells were used in all studies.

#### Jurkat cells

MART-1 Jurkat cells recognizing HLA-A2/MART-1 tetramer and negative control Jurkat cells were kindly provided by Ray Zane and Judi Baker (Beckman Coulter Immunomics, San Diego, CA).

Frozen PBMC Samples: Frozen peripheral blood mononuclear cells (PBMCs), screened HIV negative, were used in this study. PBMC from blood of HLA-A2 healthy subjects (AllCells, LLC, Emeryville, CA and American Red Cross) were isolated using Ficoll gradient centrifugation method. Cells were stored at -120°C and freshly thawed for analysis following standard procedures. PBMC was used as negative matrix in TIL cell spiking studies and also serve as antigen presenting cells (APC) in real time RT-PCR and ELISPOT analysis. Proof of principle studies were performed using frozen PBMC from three melanoma patients (kindly provided by Dr. Francesco Marincola, NCI, NIH, Bethesda, Maryland).

##### Patient PBMC samples

Frozen PBMC from the fourth melanoma patient which demonstrated immunologic response is also included as an example; samples from this patient are part of the clinical testing to monitor cancer vaccine potency of a Phase I/II clinical trial conducted by Genzyme Corporation.

### Antibodies, peptides, tetramers, oligonucleotides, and other critical reagents

#### Antibodies

The following antibodies and reagents were used: anti-CD8-FITC (BD Bioscience, San Jose, CA), anti-human IFNγ (Pharmingen, San Diego, CA), biotinylated anti-human IFNγ (Pharmingen),

#### Peptides

HLA-A2 (*0201) restricted peptides for gp100 included peptides beginning with amino acid (aa) number 154, 209 (native or 210M-modified), 280, 457, and 476. HLA-A2 restricted antigenic peptide for MART-1 included peptide 26–35 (native)/26–35 (27L, modified). The peptides were synthesized by New England Peptides, Inc. (Gardner, MA) and their aa sequences are shown, gp100_209 _(IDTQVPFSV), gp100 peptide pool [gp100_209_, gp100_154 _(KTWGQYWQV), gp100_280 _(YLEPGPVTA), gp100_457 _(LLOGTATLRL), and gp100_476 _(VLYRYGSFSV)], MART-1_27–35 _(AAGIGILTV), Flu (GILGFVFTL), and HIV (ILKEPVHGV). All PBMC samples were screened negative for HIV, allowing use of HIV peptide as negative controls. All peptides are HLA-A2 (Class I) restricted, therefore, CD8^+ ^T cell IFNγ response is expected upon peptide stimulation.

#### Tetramers

The following HLA-A2 (A*0201) tetramers (Beckman Coulter Immunomics, San Diego, CA) were used including Negative Control (T01044, containing a proprietary irrelevant peptide not being recognized by human TCR), gp100_209–217(210M) _(T01012, IMDQVPFSV), MART-1_26–35(27L)_(T01008, ELAGIGILTV), and Influenza-Flu (T01011, GILGFVFTL) tetramer. Modified gp100 and MART-1 tetramers with prolonged stability and high affinity were used. To minimize assay variability, tetramers used here for assay validation were from the same lot as the ones for clinical sample testing. All three tetramers (gp100, MART-1, and Negative) were assembled from the same Biotinylated HLA-A2 monomer lot and the same Streptavidin-PE lot. Stability of the tetramers was monitored using TIL cells. All tetramers contain HLA-A2 restricted peptides, therefore only CD8^+ ^T cells are expected to be detected.

#### Oligonucleotides

Oligonucleotide primers for real time RT-PCR were synthesized by Life Technologies. For IFNγ and CD8 cDNA synthesis, human IFNγ reverse transcription (RT) primer (5'-CTTTCCAATTCTTCAAAATG-3') and CD8 RT primer (5'-GACAGGGGCTGCGAC-3') were used, respectively. For Real Time RT-PCR analysis, the following primer pairs were used, human IFNγ forward primer (5'-ACGTCTGCATCGTTT TGGGTT-3')/reverse primer (5'-GTTCCATTATCCGCTACATCTGAA-3') and human CD8 forward primer (5'-CCCTGAGCAACTCCATCA TGT-3')/reverse primer (5'-GTGGGCTTCGCTG GCA-3'). Probes were synthesized by IDT for detection of IFNγ (5'-TCTTGGCTGTTACT GCCAGGACCCA-3') and CD8 (5'-TCAGCCACTTCGTGCCG GTCTTC-3').

#### Additional critical reagents

Streptavidin-Alkaline Phosphatase (Pharmingen) for ELISPOT; PHA (Sigma, St Louis, MO) as positive controls for real time RT-PCR and ELISPOT; Qiagen Rneasy Mini Kit (74106, Qiagen), Promega Reverse Transcription Kit (A3500, Promega), and TaqMan Universal Mix (4304437, Applied Biosystems) for RT-PCR.

### Equipment

FACSCalibur with CellQuest Pro software (BD Biosciences, San Jose, CA) was used for Tetramer analysis.

ABI Prism 7700 division sequence detector (Perkin Elmer/Applied Biosystem was used for real time PCR studies.

The FACSCalibur and ABI Prism 7700 division sequence detector were calibrated and maintained under GLP compliance. Analysts were trained on equipment SOPs prior to performing the studies.

Zeiss stereomicroscope (Carl Zeiss, Germany) was used for ELISPOT analysis.

Additional equipment (pipettes, balance, incubator, biosafety cabinet, centrifuge, freezer, and refrigerator, etc) were all calibrated and maintained under GLP compliance.

### Tetramer assay

The tetramer assay was optimized prior to initiation of the validation study (data not shown). Tetramer (0.1 μg/mL) titration (2.5, 5, 10, and 20 μL) was performed and the use of 10 μL was found to be optimal. Long term performance of the tetramer was monitored to achieve optimal binding and to assure longitudinal assay performance. Tetramer binding temperature (room temperature-RT or 2–8°C) was also evaluated and RT was chosen. Co-staining with anti-CD3 showed decrease tetramer binding probably due to proximity of CD3 and TCR, therefore anti-CD3 staining was not used. Fixed cells were shown to have decreased binding as compared to fresh. Therefore, freshly thawed, unfixed PBMC were used for validation study and clinical sample testing.

Since there is a very low percentage of gp100 and MART-1 tetramer positive cells in healthy subjects, TIL cells were used for method validation studies. TIL1520 (gp100 specific) or TIL1143 (MART-1 specific) at 1–5 × 10^4 ^cells/100 μL/tube were stained in FACS buffer (PBS without Ca^2+ ^and Mg^2+^, 1% BSA, 0.1% Sodium Azide) with 10 μL of tetramer-PE (0.1 μg/μL) and 10 μL of anti-CD8-FITC at room temperature (RT) for 1 hour in a 23–25°C incubator. Cells were washed with 3 mL of FACS buffer and harvested by centrifugation at 290 g (1500 rpm) for 7 minutes. Cells were re-suspended in 0.5 mL of FACS buffer. Ten μL of Propidium Iodide (PI) was added before acquisition for viable cell gating. Total of 10,000 to 20,000 TIL cells (un-gated events) were acquired. For frozen PBMC analysis, same staining procedure was used except that a total of 10^6 ^freshly thawed cells were stained and 500,000 cells were acquired. Data was analyzed using Cell Quest Pro Software. Percent tetramer positive cells among viable CD8^+ ^cells were shown in quadrant statistics from CD8-FITC vs. Tetramer-PE dot blot. Viable CD8^+ ^cells were defined by simultaneous gating on the triple regions, region 1 (lymphocytes from FSC vs. SSC), region 2 (viable cells-PI negative cells from FSC vs. PI), and region 3 (CD8+ cells from FSC vs. CD8). Assay validation was performed under GLP and following the method SOP.

**Figure 1:**
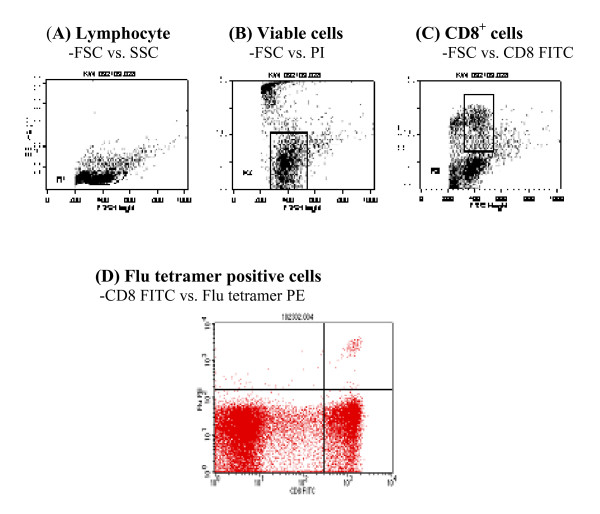
**Detection of tetramer positive cells among PBMC**. Gating sequence is shown in the upper panel. (A) R1-Lymphocyte gate, FSC (x-axis) vs. SSC (y-axis). (B) R2-Viable cell gate, FSC (x-axis) vs. PI (y-axis). (C) R3-CD8^+ ^cell gate, FSC (x-axis) vs. CD8 FITC (y-axis). Flu-tetramer positive cells are shown in (D) Flu tetramer positive cells, CD8 FITC (x-axis) vs. Flu tetramer PE (y-axis), gated on R1 and R2 for viable lymphocyte. CD8 negative cells are shown (with R3 off), demonstrating assay specificity.

As an example, Flu tetramer binding to frozen PBMC from a HLA-A2 healthy subject is shown in Figure 1, including gating sequence (A) lymphocyte-FSC vs. SSC, (B) viable cells (PI negative)-FSC vs. PI, and (C) CD8^+ ^T cells-FSC vs. CD8 FITC. Tetramer positive cells are illustrated in (D) on gated viable lymphocytes-CD8 FITC vs. Flu Tetramer PE gated on viable lymphocytes, CD8 negative cells that lack tetramer binding are also shown.

### IFNγ real time PCR assay

Freshly thawed HLA-A2 PBMCs at 10^6 ^cells/mL/well, duplicate wells in 24-well plate, were cultured for 2 hours at 37°C with 5% CO_2 _and 95% humidity in serum free medium (AIM-V, GIBCO/BRL) stimulated with gp100_209_, gp100_pool_, MART-1, Flu, PHA (positive control), or HIV (negative control). Peptides were used at 10 μg/mL/well for gp100_209_, gp100_pool_, MART-1, Flu, or HIV. TIL1520 (gp100 specific) and TIL1235 (MART-1 specific) spiked into PBMC at various cell numbers were used as positive controls. After stimulation, cells were harvested and RNA prepared following Qiagen RNA extraction protocol. RNA was stored at <-60°C until use. RNA was thawed and concentration and purity were determined by spectrophotometer at wavelength A_260/280 _(OD_260_/OD_280 _ratio). Synthesis of cDNA was done following manufacturer's protocol (Promega) using AMV Reverse Transcriptase with 25 μM of RT primer for IFNγ or CD8. Samples were stored at -15°C until further analysis.

Real Time RT-PCR analysis was performed using forward and reverses primer (each at 25 μM) for IFNγ or CD8. The probes were used at 0.2 and 0.3 μL for IFNγ and CD8, respectively.

Positive control cDNA (IFNγ and CD8 plasmid, Invitrogen) were run in duplicate at various concentrations to generate standard curves for IFNγ and CD8. Copy numbers for IFNγ and CD8 was determined.

For clinical data analysis, ratio of IFNγ over CD8 copy numbers (IFNγ/CD8) upon stimulation with gp100_209_, gp100_pool_, MART-1, Flu, or PHA (a positive control) was compared with the ratio from HIV stimulation (negative control). Data was analyzed using mRNA copy number fold increase, defined as [(IFNγ/CD8) _gp100, MART-1, Flu, or PHA_/(IFNγ/CD8) _HIV_].

### IFNγ ELISPOT analysis

ELISPOT 96-well plates (MIP-S4510, Millipore) were coated with 100 μL of anti-human IFNγ antibody at 10 μg/mL in Carbonate buffer (Poly Sciences) overnight at 2–8°C. Plates were washed, blocked with PBS containing 2.5% BSA (2.5 g/100 mL) for 1–2 hours at 36–38°C in an incubator with 5% CO_2 _and ~95% humidity, and washed a second time prior to use.

Freshly thawed PBMC alone or TIL cell [TIL1520 (gp100 specific) or TIL1235 (MART-1 specific)] spiked at different levels into PBMC (4 × 10^5 ^cells/100 μL/well, PBMC High) were used. Due to the limited supply of clinical samples, the assay was also validated using a lower concentration of PBMC (10^5^/100 μL/well, PBMC Low). In this assay, freshly thawed patient PBMC (10^5^/100 μL/well) was used. Cells were cultured in triplicate wells for 24 hours at 36–38°C with 5% CO_2 _and 95% humidity in AIM-V media with Penicillin and Streptomycin. Peptides were added at 10 μg/mL including gp100_209_, gp100_pool_, MART-1_27–35_, Flu, or HIV. PHA was used as positive control.

Following culture, the cells were discarded and plates were washed with PBS. Biotinylated anti-human IFNγ was added at 100 μL/well (1.5 μg/mL, Pharmingen) and plates were incubated for 2 hours at room temperature (in a 22–26°C incubator). Plates were washed and 100 μl of Strepavidin-Alkaline Phosphatase (Pharmingen)at 1:1000 dilution was added. Plates were incubated for 30 minutes at room temperature and washed. Substrate BCIP/NBT (KPL) was added following the manufacturer's protocol and spots were allowed to develop for approximately 4 minutes or until spots were visible. The reaction was stopped with dH_2_O. Plates were dried overnight in the dark and IFNγ secreting cells (spots/well) were counted under a dissecting microscope with a video monitor. Data was analyzed using average spot number/well/10^5 ^cells, PBMC Low (or 4 × 10^5^, PBMC High) from triplicate wells. The final data was presented as number of IFNγ secreting cells (stimulated with gp100_209_, MART-1_27–35_, gp100_pool_, Flu, or PHA) – IFNγ secreting cells (stimulated with HIV as negative control).

### Statistical analysis

Tetramer flow cytometric analysis was performed using Cell Quest Pro software (BD Biosciences) and % tetramer positive cells were obtained from quadrant statistics among gated viable CD8^+ ^T cells.

IFNγ Real Time PCR analysis was done using ABI Prism 7700 software for mRNA quantification.

Additional statistical analysis was performed to examine assay accuracy and precision using Microsoft Excel. Accuracy was assessed by % Recovery, (detected value/expected reference value) × 100. Precision was examined using % CV (coefficient of variation), (SD/Mean) × 100. Linearity of Dilution (linear regression analysis) was performed using GraphPad Prism 4 (Version 4.02). Regression analysis of post-vaccine immunologic response in the representative melanoma patient was performed using JMP 7 software.

## Results

### Part 1: Tetramer assay validation

#### Specificity

Specificity (Selectivity) is the ability of an analytical method to differentiate and quantify the analyte in the presence of other components in the sample.

**Figure 2:**
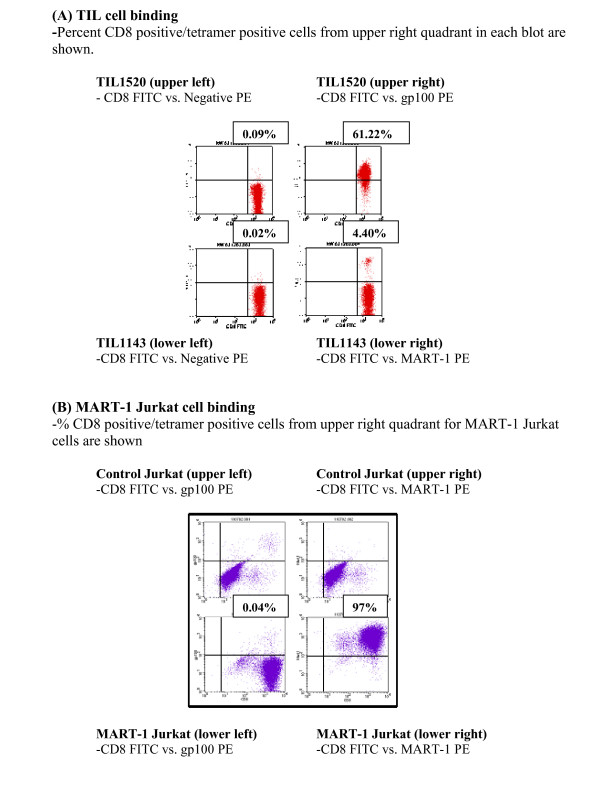
**Tetramer assay specificity**. (A) TIL cell binding: % tetramer positive cells are shown based on data in the upper right quadrant from each of the 4 blots. TIL1520 (top panel) were stained with negative tetramer (left) and gp100 tetramer (right). TIL1143 (bottom panel) were stained with negative tetramer (left) and MART-1 tetramer (right). (B) MART-1 Jurkat cell binding: % tetramer positive cells are shown based on data in the upper right quadrant from MART-1 Jurkat cell blots (lower panel) stained with irrelevant gp100 tetramer (left) or relevant MART-1 tetramer (right). Control Jukat cells (upper panel) were stained with both tetramers (% tetramer positive cells are <0.05%, data not shown).

Tetramer assay specificity is defined as TIL cells which lack binding to negative tetramer and irrelevant tetramer and show specific binding to the relevant tetramer (TIL1520 binding to gp100 and TIL1143 binding to MART-1). Low background binding was observed from cells with no tetramer (0.00% for TIL1520 and 0.02% for TIL1143, data not shown) or stained with the negative tetramer (0.09% for TIL1520 and 0.02% for TIL1143), Figure 2(A). Tetramer binding specificity is demonstrated, Figure 2(A); the gp100 tetramer showed specific binding to TIL1520 cells (61.22%) and not TIL1143 cells (0.06%, data not shown); similarly, MART-1 tetramer bound specifically to TIL1143 (4.40%) and not TIL1520 cells (0.19%, data not shown).

Unlike the high percentage of binding of gp100 tetramer to TIL1520, MART-1 tetramer binding to TIL1143 was at a much lower percentage probably due to activation associated TCR down modulation on TIL1143 (data not shown). To confirm that MART-1 tetramer can maximally detect all of the MART-1 specific T cells under the assay conditions used, Jurkat cells that were genetically modified to express TCR that recognizes MART-1/HLA-A2 (generously provided by Judi Baker and Ray Zane, Beckman Coulter Immunomics, San Diego, CA) were used and 97% of MART-1 tetramer positive cells were detected; irrelevant gp100 tetramer binding to the MART-1 Jurkat cells was minimal (0.04%), Figure 2(B). Control Jurkat cells did not show binding to MART-1 tetramer while there was some background binding to the gp100, Figure 2(B). Due to the following acquisition sequence (MART-1 Jurkat/gp100, MART-1 Jurkat/MART-1, Control Jurkat/gp100, and Control Jurkat/MART-1), we believe that carry over of the MART-1 Jurkat/MART-1 tetramer sample caused background staining in Control Jurkat/gp100 tetramer. This experiment could not be repeated due to an insufficient number of cells.

#### Accuracy

The accuracy of an analytical method describes the closeness of mean test results (detected) obtained by the method to the true value (expected) of the analyte. Accuracy was assessed by percent recovery [(detected value/expected value) × 100] and 80–120% is considered acceptable.

Due to the lack of true value from a standard reference material for the tetramer assay and lymphocyte phenotype analysis using flow cytometric methods in general, our attempt at assessing accuracy was unsuccessful. We used detected data values from undiluted TIL cells to establish reference true value for the diluted samples (by multiplying the dilution factor); % tetramer positive cells detected especially at the low level, were found to be outside of 80–120% of the reference value, data not shown. TIL cells showed tetramer binding variability due to culture conditions and cell passages; this variability makes establishing a true value using detected values from undiluted samples challenging.

To monitor long term assay performance, we generated TIL1520 and TIL1143 working cell banks stored in liquid N_2 _in a single using aliquot and used freshly thawed cells (no additional cell culture) as assay quality control material. (data is shown under precision-long term inter-assay performance assessment).

#### Precision

The precision of an analytical method describes the closeness of agreement (degree of scatter) between a series of measurements obtained from multiple sampling of the same homogenous sample under the prescribed conditions.

Intra assay precision (repeatability) expresses the precision under the same operating conditions over a short interval of time (in a single assay). Intra assay precision is determined by % CV (coefficient of variation) as (SD/Mean) × 100 tested multiple times by one analyst in a single assay. Inter assay precision (Intermediate Precision) is defined as the variability of a sample (% CV) tested in multiple assays on more than one day. For example, factors that contribute to inter assay variability for the tetramer assay include cell preparation, staining methods, machine setting, gating during acquisition and data analysis. Percent CV <20% is considered acceptable for analytical assays in general. For flow cytometry assays to detect cells at a very low level, a higher %CV is expected. Since a low frequency of tetramer positive cells is expected among patient PBMC, using a high percentage of gp100 tetramer positive cells among TIL1520 is not suitable for assessment of assay precision at the low level. TIL1520 was also spiked into the negative population (TIL1520 stained with the negative tetramer) to generate two samples containing a low percentage of gp100 tetramer positive cells (Low 1 and Low 2) for assessment of assay precision. Undiluted TIL cells were included as a high control (High).

Intra assay precision (% CV) for both gp100 and MART-1 tetramer are acceptable (<20% CV). Representative data is shown in Table 1. Precision for gp100 tetramer showed precision of 2%CV using undiluted TIL1520 (High). Percent CV was 16 and 10% when TIL1520 were further diluted to generate samples with a lower percentage of tetramer positive cells. For MART-1, % CV is 6%.

**Table 1:** Tetramer assay precision

	**Tetramer**	**gp100**	**MART-1**
	**Cells**	**TIL1520**	**TIL1143**

**Intra assay**			

High	Range	54.48–57.21	3.33–3.96

	Mean (n = 5)	56.15	3.64

	SD	1.14	0.23

	%CV	2	6

			

Low 1	Individual Value	1.05, 1.32	

	Mean (n = 2)	1.19	

	SD	0.19	

	%CV	16	

			

Low 2	Individual Value	0.52, 0.60	

	Mean (n = 2)	0.56	

	SD	0.06	

	%CV	10	

			

**Inter assay**			

High	Range	41.86–62.63	3.26–4.65

	Mean (n = 5)	53.38	3.74

	SD	9.44	0.55

	%CV	18	15

			

**Long term**			

High	Range	62.69–99.49	1.45–7.75

	Mean (n = 48)	93.24	4.10

	SD	6.48	1.85

	%CV	7	45

Data is shown as % Tetramer positive cells (Range, Mean, n, SD, and %CV). Cells are used as undiluted (High) or diluted in the negative population (Low 1 and Low 2 for TIL1520). Long term assay precision (inter assay) is shown using data collected in 48 tests.

Inter assay precision (% CV) for gp100 was 18% and MART-1 was 15%, and therefore both met the validation criteria (<20%), Table 1. Analyst variability (%CV) between 2 operators is 12% (gp100) and 20% (MART-1); equipment shut down/re-start variability (% CV = 2% for MART-1) was minimal (data not shown). Due to high assay variability inherent in flow cytometric methods and the low level of tetramer positive cells (expected in patients), we a designed clinical testing regimen to minimize assay variability. In this testing regimen, frozen longitudinal PBMC samples from each patient were tested in a single assay by a single operator.

TIL cells maintained in culture at different passages experience variation in TCR expression level which could contribute to variability in the tetramer assay. To monitor long term assay performance, a working cell bank was prepared for each line (TIL1520 and TIL1143) and cells were frozen in single use aliquots. Freshly thawed cells (without additional culturing) were analyzed in each assay for clinical sample testing, serving as quality controls. This practice allows us to analyze long term (2 year) inter-assay precision (February 2003 to May 2005) which was not feasible during assay validation. Precision (%CV) from 48 assays performed by three different operators showed that gp100 tetramer analysis had acceptable %CV (7%), Table 1. MART-1 tetramer analysis variability was high with % CV of 45%, probably due to the low level of tetramer positive cells in combination with the high inter-assay variability that is expected in flow cytometric methods. This finding supported our clinical testing regimen; all longitudinal frozen PBMC samples from each patient were tested in a single assay by a single operator, allowing assessment of vaccine potency compared to pre-treatment baseline values in each patient.

#### Spike and recovery

Assessment of spike and recovery of an analyte in biological matrix (matrix effect) is defined as the direct or indirect alteration or interference in response due to the presence of unintended analytes or other interfering substances in the sample.

Due to the lack of a standard reference material to establish a true value, recovery (% tetramer positive cells detected) could not be assessed. In addition, the TIL cells showed unexpected FSC vs. SSC properties. Compared to resting T cells among PBMC, TIL cells resembled activated lymphocytes. (lymphocyte blasts). The use of a single gate to analyze the mixed cell population (TIL spiked in PBMC) was also found to be challenging (data not shown). Although TIL cells have the same HLA-A2 allele as the PBMC used here, the non-A2 alleles are expected to be different for other HLA loci (DR and DQ, for example), which could result in cell-cell interaction (aggregation).

#### Limit of detection (LOD) and limit of quantification (LOQ)

LOD is defined as the lowest concentration of an analyte that the bioanalytical procedure can reliably differentiate from background noise.

LOQ is defined as the lowest amount of an analyte in a sample that can be quantitatively determined with suitable precision and accuracy.

Due to the lack of a standard reference material to establish a true value, LOQ was not examined for the tetramer assay. Assay LOD and sensitivity was examined.

MART-1 (27L) tetramer is known to be recognized by CD8^+ ^T cells in healthy subjects, therefore, % MART-1 tetramer positive cells in normal PBMC samples (endogenous level), shown in distribution study (Table 2), could not be used to assess background signal. Low % positive cells were detected among 20 PBMC samples using the negative control tetramer and gp100 tetramer, 0.11% and 0.07%, respectively (Mean value from 20 samples, described in Normal Distribution studies). At such low level, assay variability is expected to be higher and SD was found to be 0.11% (negative tetramer) and 0.09% (gp100). It is not a common practice in the field to use the negative control tetramer binding to establish assay background noise level; most laboratories use values from unstained cells. Our data showed that unstained cells had 0% tetramer positive cells in most cases. However, on occasion, positive cells were found with values less than 0.06% (data not shown).

**Table 2:** Normal distribution, tetramer binding among 20 healthy subjects

**Donors**	**Negative**	**gp100**	**MART-1**
1	0.07	0.07	0.42

2	0.04	0.02	0.49

3	0.02	0.02	0.47

4	0.05	0.02	0.43

5	0.04	0.02	0.54

6	0.12	0.02	0.59

7	0.03	0.04	0.40

8	0.13	0.02	0.58

9	0.24	0.07	0.48

10	0.07	0.06	0.63

11	0.04	0.07	0.82

12	0.07	0.02	0.55

13	0.11	0.05	0.39

14	0.03	0.02	0.63

15	0.10	0.04	0.39

16	0.06	0.02	0.26

17	0.03	0.06	0.35

18	0.06	0.24	ND

19	0.40	0.35	1.15

20	0.40	0.25	0.82

Mean	0.11	0.07	0.55

SD	0.11	0.09	0.21

Range	0.02–0.40	0.02–0.35	0.21–1.15

ND, not determined due to insufficient cells.

% Tetramer positive cells for negative tetramer, gp100, and MART-1 are shown.

Assay sensitivity can be improved by collecting a larger number of events on the cytometer. Due to the limited supply of TIL cells and clinical PBMC samples from patients and the need for reasonable assay throughput/turn around time to maintain cell viability during acquisition, we evaluated total acquisition events vs. cell quality (viability by PI and % tetramer positive cells). Our data supported collection of 10,000–20,000 TIL cells and 200,000–500,000 PBMC. To further assess assay sensitivity under our assay condition, we spiked Flu positive donor PBMC at various percentages (100, 50, 25, 12.5, 6.3, 3.1, and 0) into the negative PBMC (unstained cells from the same donor) and % Flu tetramer positive cells were analyzed from total of 200,000 events collected. At the lowest level assessed (3.1% Flu positive PBMC among negative PBMC), Flu tetramer positive cells were detected in 2 tests at 0.022 % (1/4545) and 0.015 (1/6667). We expect that with increased total acquisition events, our assay sensitivity could reach the level found by other laboratories (0.01–0.0125%, equivalent to 1/8000–1/10,000). Studies were also performed using TIL1520 spiked into TIL1143 stained for gp100 and TIL1143 spiked into TIL1520 stained for MART-1. Assay sensitivity was 1/1000 to 1/2000 due to the lower number of events (10,000) collected. We believe our assay sensitivity is equivalent to the level found by other laboratories. Due to limited volume of samples collected in melanoma patients, we were limited to acquiring the number of events as described in this manuscript.

#### Calibration standard curve and linearity of dilution

Due to the lack of a standard reference material and knowing that TIL cells have different binding characteristics (affinity, specificity, etc) compared to patient PBMC, a calibration standard curve was not used to quantify tetramer positive cells.

The highest % tetramer positive cells were detected using undiluted TIL cells. TIL cells were further diluted into the negative cell population to assess assay linearity.

**Figure 3:**
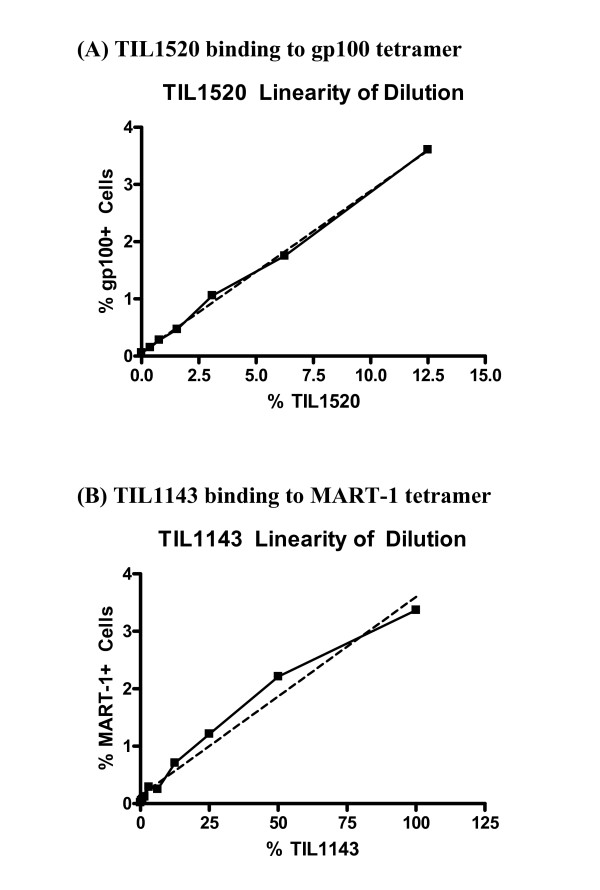
**Tetramer assay linearity of dilution**. (A) TIL1520 binding to gp100 tetramer. Correlation of % TIL1520 used (x-axis) vs. % gp100 tetramer positive cells detected (y-axis) is shown. (B) TIL1143 binding to MART-1 tetramer. Correlation between % TIL1143 used (x-axis) vs. % MART-1 tetramer positive cells (y-axis) is illustrated.

TIL1520 cells (gp100 positive) were spiked into a negative population at 12.5%, 6.25%, 3.1%, 1.56%, 0.78%, 0.39%, and 0% (x-axis) and %gp100 positive cells (y-axis) were analyzed. Sample dilution linearity is shown in Figure 3(A). TIL1520 cell dilution (x) vs. % gp100 positive cells (y) showed good correlation (r^2 ^0.9977, y = 0.28× + 0.06), using linear regression analysis. Similarly, TIL1143 cells (MART-1 positive) were spiked into a negative population at 100, 50, 25, 12.5, 6.25, 3.1, 1.56, 0.78, 0.39, and 0% (x-axis) and the % MART-1 tetramer positive cells (y-axis) were analyzed. TIL1143 cell dilution linearity is shown in Figure 3(B), also with good correlation (r^2 ^0.9754, y = 0.04× + 0.14). Compared to TIL1520 (gp100), a lower degree of linearity was observed for TIL1143 (MART-1). Dashed line illustrates the best fit from linear regression analysis.

#### Sample stability

Sample stability was assessed and a summary is described here (data not shown). Short-term stability (room temperature and 2–8°C) was poor for both fresh blood (<48 hour) and PBMC (<24 hour); such storage is not recommended. Clinical blood samples were processed at the site upon collection using the Ficoll gradient method for PBMC isolation. The PBMC were then cryopreserved and stored in liquid nitrogen (LN_2_) until shipment to Genzyme (on dry ice). Upon thawing, long term stability (LN_2_, -120°C) was evaluated using trypan blue exclusion and by additional T cell functional analysis (proliferative response to mitogen PHA using ^3^H-TdR incorporation). Frozen PBMC were found to be stable for at least 5 years and we continue to evaluate the stored PBMC samples over time. Freeze/thaw stability is limited to 1 cycle, which is well-documented. Freshly thawed samples were analyzed immediately in Tetramer, Real time RT-PCR, and ELISPOT assays.

PBMC stability for real time RT-PCR and ELISPOT will not be discussed separately.

#### Normal distribution

HLA-A2 PBMCs from 20 healthy subjects were tested in the tetramer assay to define normal distribution (Table 2). Among 20 normal individuals, binding to negative tetramer (0.11%) and gp100 (0.07%) was low. Higher MART-1 (27L) binding (0.55%) was observed. MART-1 tetramer is known to be cross-reactive in healthy PBMC samples, described previously by Pittet et al. [13]. MART-1 positive cells detected in normal PBMC samples were found to have low MFI (median fluorescent intensity), in contrast to MART-1 positive cells detected in TIL1143. It is difficult to distinguish MART-1 positive cells with low MFI from the negative cells and the percent is largely dependent on quadrant position. Therefore, defining the tetramer positive cell population in patients cannot rely solely on the percentage of positive cells especially those with low MFI. Identification of a distinct population, well separated from the negative population, and with high MFI is also important.

#### Determining reference ranges for assay controls

Assay controls consisted of single use aliquots of TIL1520 (gp100 control) and TIL1143 (MART-1 control) working cell banks stored frozen in LN_2_. Freshly thawed longitudinal PBMC samples from each patient were analyzed for gp100 and MART-1 tetramer binding in a single assay using these positive controls. Data from TIL controls was compared to historical data. Negative control tetramer binding to TIL cells and PBMC was also used as negative controls.

PBMC viability (>80% viable by trypan blue exclusion after thaw) and PI exclusion during flow cytometry data analysis were additional cell quality controls.

### Part 2: IFNγ real time RT-PCR validation

#### Specificity

IFNγ real time RT-PCR specificity is defined as lack of response to irrelevant peptides and HIV negative control peptide and positive response to relevant peptide stimulation (TIL1520 with gp100 peptides and TIL1235 with MART-1 peptide).

**Figure 4:**
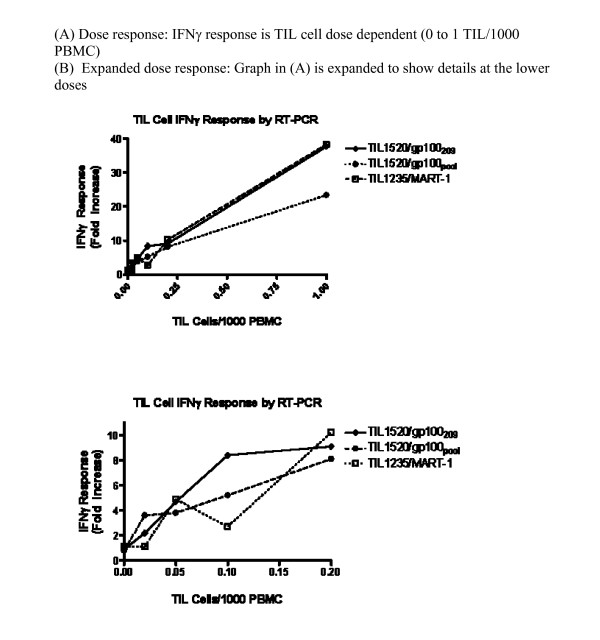
**IFNγ real time RT-PCR specificity**. TIL cells at different numbers were spiked into 10^6 ^PBMC; response (Fold Increase over HIV, normalized by CD8 copy numbers) vs. TIL cell frequency is shown. (A) Full TIL dose range (0 to 1 TIL/1000 PBMC) and (B) Response at lower dose range (0 to 0.2 TIL/1000 PBMC)

The real-time RT-PCR assay showed a high level of specificity through the validation process. HLA A2 PBMC alone from healthy subjects did not show response to melanoma peptides; a dose dependent IFNγ response, fold increase (IFNγ relevant peptide/CD8)/(IFNγ _HIV_/CD8), was only seen in PBMC with spiked TIL cells stimulated with relevant peptide, TIL1520 stimulated with gp100_209 _and gp100_pool _and TIL1235 stimulated with the MART-1 peptide (Figure 4). As expected, these TIL cells did not respond to the irrelevant peptide (data not shown) or the negative control (HIV) peptide. The positive control PHA response produced consistently high IFNγ expression levels indicating cell viability and expected cell function (described later in Spike and recovery, LOD  and LOQ, and Normal distribution studies). Variability was observed among individual donors, which was probably due to differences in % CD8^+ ^T cells and antigen presenting cells as well as cell functionality. A complete data set will be shown and discussed in normal distribution studies.

#### Accuracy and precision

The real time RT-PCR assay was examined for assay accuracy and precision by spiking 1000 copies of IFNγ plasmid per sample in 80 repeats (n = 80) for intra-assay and 18 repeats (n = 18) for inter-assay performance characteristics. Two analysts performed the analysis. Assay was found to be both accurate and precise with % recovery between 80–120% (analyst 2 had a 123%) and % CV < 20%, respectively (Table 3).

**Table 3:** IFNγ real time RT-PCR accuracy and precision

	**Analysts 1**	**Analysts 2**
**Intra Assay (n = 80)**		

Expected Value	1000	1000

Detected Value (Mean)	954	1233

SD	109	216

Precision (% CV)	11.4%	17.5%

Accuracy (% Recovery)	95%	123%

		

**Inter Assay (n = 18)**		

Expected Value	1000	1000

Detected Value (Mean)	1100	1133

SD	14	65

Precision (% CV)	10.3%	5.8%

Accuracy (% Recovery)	110%	113%

Data is shown as IFNγ gcopy numbers determined by 2 analysts to assess accuracy and precision for intra assay (a single assay with 80 repeats) and inter assay (in 18 inter day runs). Expected value is the copy number used as PCR template. Accuracy is examined using % Recovery (Detected/Expected) and precision is examined using %CV (SD/Mean).

#### Calibration standard curve and linearity of dilution

**Figure 5:**
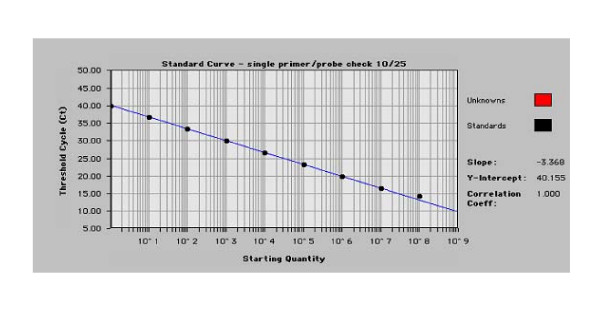
**IFNγ real time RT-PCR standard curve (linearity)**. Linear response (IFNγ plasmid copy number vs. Ct) is shown. Curve characteristics are also indicated.

A standard curve was run using plasmid (10 to 10^8 ^copies, 1:10 serial dilution) and no-template controls (Figure 5). Linearity was determined by using a standard curve (starting quantity vs. threshold cycle-Ct) generated using plasmid IFNγ at 10–10^8 ^copies. Linear amplification of log serial dilutions was observed with Slope (-3.368), Y-intercept (40.155), and Correlation Coefficient (1.000). Standard curve was determined on 6 TaqMan plates and no significant differences were found.

#### Spike and recovery

TIL 1520 and TIL1235 spiked in HLA A2 PBMC (from 10 healthy subjects) and stimulated with peptides were used to further assess real time RT-PCR assay performance characteristics. Dose (number of TIL cells) dependent IFNγ response was observed (Table 4). IFNγ response, [(IFNγ/CD8)_peptideorPHA_/(IFNγ/CD8)_HIV_], correlated with increased number of TIL cells spiked. TIL1520 responded to gp100 peptides, Table 4(A) and TIL1235 responded to MART-1 peptide, Table 4(B). Response to HIV, Flu, and PHA was also observed as expected. HIV response was low in all donors. Flu and PHA response vary among different individuals, which may due to difference in number of CD8^+ ^T cells and antigen presenting cells, as well as cell function.

**Table 4:** Real time RT-PCR spike and recovery and normal distribution: IFNγ response from TIL cells spiked in normal PBMC

(A) TIL1520 response to gp100 peptides								
	Flu		**gp100_209_**		**gp100_pool_**		PHA	

**TIL1520/PBMC**	Mean	SD	Mean	SD	Mean	SD	Mean	SD

PBMC only	40.1	95.0	0.9	0.4	1.1	0.4	337.4	316.4

1/50000	56.7	148.0	4.0	4.2	2.8	1.6	261.9	238.9

1/20000	33.5	76.4	16.1	18.3	8.9	8.2	347.3	439.2

1/10000	40.0	100.4	14.1	8.6	34.5	65.7	168.4	163.8

1/5000	40.9	99.9	24.2	21.4	21.4	15.0	258.8	227.3

1/1000	25.9	63.4	55.2	30.3	49.8	36.9	126.1	95.8

								
(B) TIL1235 response to MART-1 peptide								

	Flu		**MART-1**		PHA			

**TIL1235/PBMC**	Mean	SD	Mean	SD	Mean	SD		

PBMC only	28.2	51.7	1.1	0.5	366.3	516.5		

1/50000	36.1	101.3	1.8	1.1	162.2	142.6		

1/20000	56.8	113.8	3.1	1.8	161.1	145.1		

1/10000	58.7	125.1	3.7	1.8	183.7	197.9		

1/5000	41.3	86.3	5.2	2.2	163.6	199.1		

1/1000	46.1	98.0	17.8	12.3	168.1	221.7		

TIL cells at different numbers were spiked into PBMC from individual healthy donors and IFNγ response examined. Response (average from 10 different donors) is shown as fold increase. All donors are HLA-A2 positive screened and confirmed to be HIV negative. Peptide stimulation is HLA-A2 restricted and specific for CD8^+ ^T cells. The SD is high due to variability in individual response among 10 healthy subjects. This finding is expected. Fold increase is calculated as follows using CD8 as internal controls: (IFNγ from peptide and mitogen stimulation/CD8)/(IFNγ from HIV stimulation/CD8).

#### LOD and LOQ

LOQ and LOD were determined by spiking IFNγ plasmid and internal control CD8 plasmid at various copy numbers (1 to 10^5^). Each sample was measured in 12 repeats and assay results were summarized in Table 5. LOQ for both IFNγ and CD8 is determined as 1000 copies where quantification was achieved with acceptable accuracy (% Recovery within 80–120%) and precision (% CV < 20%).

**Table 5:** IFNγ real time RT-PCR LOQ and LOD

Expected copies	Detected copies (Mean, n = 12)	SD	Number of positive results/total 12 tests	% Recovery	%CV
**IFNγ**			12/12		

100,000	104,508	15,676	12/12	105%	15%

10,000	9,032	1,174	12/12	90.3%	13%

1,000	942	198	12/12	94.2%	21%

100	80	27.2	12/12	80%	34%

10	10	NA	8/12	NA	NA

1	1	NA	3/12	NA	NA

					

**CD8**					

100,000	93,334	8,400	12/12	93%	9%

10,000	10,533	2,001	12/12	105%	19%

1,000	1,035	134	12/12	103%	13%

100	109	41.4	12/12	109%	38%

10	14	NA	10/12	NA	NA

1	2	NA	3/12	NA	NA

NA, not applicable (spiked copy number at 10 and 1 did not show detection in all 12 tests, therefore SD, % Recovery, and %CV is not analyzed).

IFNγ plasmid at different copy numbers were used for assessment of LOQ and LOD. Expected and detected values are shown. % Recovery (Detected/Expected) and % CV (SD/Mean) are calculated to assess assay accuracy and precision, respectively.

LOD for IFNγ and CD8 is 100 copies where all 12 repeats tested positive above the background.

LOD for gp100 and MART-1 specific IFNγ response was further assessed using TIL1520 and TIL1235 spiked in PBMC, also described in normal distribution studies (Table 4).

LOD was determined as 1/50,000 cells where IFNγ response was detected above the HIV control (fold increase of 1.0) and PBMC only (no TIL spiked).

#### Normal distribution

Normal distribution of real time RT-PCR (PBMC only, no spiked TIL cells) is shown in Table 4. Average IFNγ response (fold increase) to gp100 (209 and pool) and MART-1 from healthy subjects (n = 10) is <1.1.

### Part 3: IFNγ ELISPOT validation

This assay was first validated using 80 TIL cells spiked into 4 × 10^5 ^PBMC per well (96 well plate), designated as High PBMC Assay. Due to the limited volume of blood collected from clinical melanoma patients, we also validated the assay using a lower number of PBMC (80 TIL cells spiked into 10^5 ^PBMC/well), designated as Low PBMC Assay. Peptide concentrations remained the same. Compared to the Low PBMC Assay, IFNγ secreting cells among the same number of TIL cells were found to be slightly higher in the High PBMC Assay, probably due to a higher number of antigen presenting cells in the PBMC population.

Data presented here are from the low PBMC assay except in LOD and LOQ; data from both high and low PBMC assays are shown.

#### Specificity

ELISPOT specificity is defined as the lack of response to irrelevant peptides and HIV peptide together with a positive response to relevant peptide stimulation (TIL1520 with gp100 peptides and TIL1235 with MART-1 peptide).

To evaluate assay specificity, a total of 80 TIL cells were spiked into PBMC (10^5 ^cells/well) and the number of IFNγ secreting cells following peptide stimulation was examined. Two analysts, each using two PBMC lots, performed five assays each. Data from two PBMC lots were comparable and variability between the two analysts was low. Data from PBMC lot 1 by analyst one is shown in Table 6. Among TIL1520, IFNγ secreting cells/well (average from triplicate wells), were detected upon gp100_209 _stimulation at an average of 41 secreting cells/well. Stimulation with gp100 _pool _containing gp100_209 _did not result in an increased frequency of IFNγ secreting cells (39 cells/well) compared to gp100_209 _alone, confirming that the TIL1520 is gp100_209 _specific. This is consistent with the real time RT-PCR findings (described in Part 2). Similarly, IFNγ secreting cells were detected among TIL1235 following MART-1 peptide stimulation at an average of 9 secreting cells/well. Lower numbers of secreting cells (<2) were detected upon irrelevant peptide stimulation, further demonstrating assay specificity. PBMC stimulated with Flu peptide showed IFNγ response at 212 IFNγ secreting cells/well while HIV response was low with 0.9 IFNγ secreting cells/well (data not shown).

**Table 6:** IFNγ ELISPOT assay specificity

**TIL**	**TIL1520**			**TIL1235**		
**TIL specificity**	**gp100_209_**			**MART-1**		

**Peptide specificity**	**Relevant**	**Relevant**	**Irrelevant**	**Relevant**	**Irrelevant**	**Irrelevant**

**Peptide**	**gp100_209_**	**gp100_pool_**	**MART-1**	**MART-1**	**gp100_209_**	**gp100_pool_**

1	44	41	1	8	2	2

2	42	31	1	9	0	1

3	37	40	1	10	2	1

4	43	32	1	10	1	1

5	38	52	2	7	1	1

Mean (n = 5)	41	39	1.1	9	1.3	1.3

SD	3.1	8.5	0.4	1.2	0.8	0.6

TIL cells (80 cells/well) were spiked in two different lots of PBMC (10^5 ^cells/well), stimulated with peptides, and analyzed for the number of IFNγ secreting cells per well (average value from triplicate wells). Two analysts each performed five assays. The numbers of IFNγ secreting cells from 2 PBMC lots by two analysts were found to be comparable. Data (secreting cells, Mean from 5 tests, and SD) from PBMC lot 1 by analyst 1 is shown as an example.

#### Precision

Cell based functional assays such as ELISPOT are expected to have high assay variability. We consider intra assay precision acceptable with % CV < 20% and inter assay precision acceptable with % CV < 25%.

Assay precision was assessed using 80 TIL cells spiked in 4 × 10^5 ^PBMC per well (High PBMC assay) and the data is summarized below. For intra assay, two analysts each tested samples in eight repeats. Average IFNγ secreting cells/well (n = 8) from 2 PBMC lots by two analysts were found to be 37–67 (TIL1520 stimulated with gp100_209_), 39–63 (TIL1520 stimulated with gp100_pool_), and 22–39 (TIL1235 stimulated with MART-1). Percent CV ranged from 8.3–17.6% for intra assay precision which is considered acceptable (%CV < 20%). Inter assay precision was examined and each analyst assessed two PBMC lots in five assays. Average secreting cells (n = 5) was found to be 41–52 (TIL1520 with gp100_209_), 37–52 (TIL1520 with gp100_pool_), 18–31 (TIL1235 with MART-1). Among 12 runs (2 PBMC lots, 2 analysts, 3 peptides), % CV from nine runs showed % CV ranged from 8.7–20.3%. Three tests had % CV > 20% including 21.3% (TIL1520/PBMC1 with gp100 pool by Analyst 1), 21.6% (TIL1235/PBMC1 with MART-1 by Analyst 2), and 22.6% (TIL1235/PBMC2 with MART-1 by Analyst 2). Inter assay precision (%CV < 25%) is considered acceptable.

Data from 80 TIL cells spiked into two PBMC lots at 10^5 ^cells (low PBMC assay) were analyzed by two analysts each performed eight intra-day assays and 10 inter-day assays. Cells were stimulated with relevant peptide (gp100_209 _and gp100_pool _to TIL1520 and MART-1 to TIL1235) and IFNγ secreting cells were examined. Data (Table 7) from PBMC lot 1 and Analyst one is shown as an example. Both intra assay (% CV < 20%) and inter assay (% CV < 20%) precision was found to be acceptable, except TIL1520 stimulated with gp100_pool _showed %CV of 20.8% Cells stimulated with irrelevant peptide and HIV had very low background signal and % CV was high, as expected.

**Table 7:** IFNγ ELISPOT assay precision

(A) Intra assay precision								
**TIL**	**TIL1520**	**TIL1520**	**TIL1235**					

**Peptide**	**gp100_209_**	**gp100_pool_**	**MART-1**					

**Peptide specificity**	**Relevant**	**Relevant**	**Relevant**					

Test								

1	34	33	19					

2	30	43	29					

3	36	35	26					

4	40	35	23					

5	45	41	25					

6	37	34	21					

7	32	34	18					

8	28	35	22					

Mean (n = 8)	35	36	23					

SD	5.3	3.6	3.4					

% CV	15%	10%	15%					

								

(B) Inter Assay Precision								

**Cells**	**TIL1520**	**TIL1520**	**TIL1235**	**PBMC**	**PBMC**	**TIL1235**	**TIL1235**	**TIL1520**

**Peptide**	**gp100_209_**	**gp100_pool_**	**MART-1**	**Flu**	**HIV**	**gp100_209_**	**gp100_pool_**	**MART-1**

**Specificity**	**Relevant**	**Relevant**	**Relevant**			**Irrelevant**	**Irrelevant**	**Irrelevant**

Tests								

1	44	41	8	195	1	2	2	1

2	42	31	9	206	1	0	1	1

3	37	40	10	198	1	2	1	1

4	43	32	10	222	1	1	1	1

5	38	52	7	240	1	1	1	2

6	38	39	10	254	1	0	2	2

7	39	54	12	278	1	1	1	2

8	49	46	13	245	0	1	1	2

9	36	32	11	226	1	2	1	4

10	38	34	8	224	1	1	1	1

Mean (n = 10)	41	40	10	229	0.9	1.2	1.3	1.6

SD	3.9	8.4	1.8	26.0	0.3	0.8	0.6	0.9

% CV	9.6	20.8	18.3	11.4	33.3	66.6	46.2	56.3

A total of 80 TIL cells were spiked into 10^5 ^PBMC per well (Low PBMC) and tested in 8 repeats (intra assay) and 10 tests (inter assay) by two analysts. Average IFNγ gsecreting cells per well (triplicate wells) upon peptide stimulation are shown. Mean, SD, and %CV is shown for PBMC 1 by Analyst 1 as an example.

#### Accuracy, spike and recovery, and LOQ

Due to the lack of a reference standard material to establish a true value, assay accuracy, spike and recovery, and LOQ were not examined.

#### Plate homogeneity

Samples loaded at different locations across a 96-well microtiter plate showed comparable results (data not shown).

#### LOD and assay sensitivity

LOD (assay sensitivity) was assessed by spiking diminishing numbers of TIL1520 and TIL1235 cells into 4 × 10^5 ^PBMC (High) or 10^5 ^PBMC (Low) per well. TIL1520/PBMC were stimulated with gp100_209 _and gp100_pool _and TIL1235/PBMC were stimulated with MART-1. The LOD was determined to be the least number of secreting cells that could be distinguished from the background (>10 cells/well) upon stimulation with relevant peptide. The acceptable level of background secreting cells was obtained from irrelevant peptide stimulation, HIV peptide stimulation and from the results of the normal distribution study (Table 8). Data from the normal distribution study showed the number of background IFNγ secreting cells (Mean + 2 SD) to be as follows: gp100_209 _(8.9), gp100_pool _(5.2), MART-1 (6.5), and HIV (6.7). Therefore, we consider background to be 10 IFNγ secreting cells/well.

**Table 8:** ELISPOT assay normal distribution

	**gp100_209_**	**gp100_pool_**	**MART-1**	**Flu**	**HIV**
1	2	3	4	15	2

2	4	2	4	5	1

3	5	4	4	3	2

4	2	2	3	3	2

5	2	2	1	3	1

6	9	5	6	8	7

7	2	2	4	5	4

8	0	1	2	241	1

Mean	3.3	2.6	3.5	35.4	2.5

SD	2.8	1.3	1.5	83.2	2.1

Mean + 2 SD	8.9	5.2	6.5	201.8	6.7

HLA-A2 PBMC (10^5 ^cells/well) from healthy donors were stimulated with peptides and the number of IFNγ secreting cells determined.

For the High PBMC assay, the LOD for gp100 was defined as the ability to detect IFNγ secreting cells at frequency of 1/20,000 (15 secreting cells/well) among TIL1520. The LOD for MART-1 is at 1/10,000 (14 secreting cells/well). The data shown in Table 9 demonstrates that the assay sensitivity from the high PBMC assay is similar to the results published by other laboratories.

**Table 9:** IFNγ ELISPOT LOD

			**TIL1520**	**TIL1520**	**TIL1235**
		**Peptide**	**gp100_209_**	**gp100_pool_**	**MART-1**

**PBMC High**	**TIL Cells/well**	**TIL/PBMC**			

	80	1/5,000	53	57	15

	40	1/10,000	27	30	**14**

	20	1/20,000	**15**	**17**	4

	8	1/50,000	3	6	0

	4	1/100,000	6	0	1

**PBMC Low**					

	200	1/500	86	84	28

	100	1/1000	52	59	19

	50	1/2000	37	38	**11**

	25	1/4000	22	31	7

	12	1/8000	**13**	**10**	3

TIL cells were spiked into 4 × 10^5 ^(High) or 10^5^PBMC (Low) per well and limit of detection was determined (IFNγ Secreting Cell Frequency). The lowest IFNγ secreting cells detected above the background is considered LOD, values shown in bold. The corresponding TIL/PBMC ratio indicates assay sensitivity.

At first glance, assay sensitivity does not appear to be as good when the lower number of PBMC was used (10^5 ^cells/well), Table 8. Although we could still detect 10–13 spots, the detection frequency was found to be 1/8000 (10 secreting cells/well) for gp100 and 1/2000 (11 secreting cells/well) for MART-1. This finding is due to the fact that the PBMC cell count is used as the denominator when calculating the detection frequency. The lower cell number in the denominator creates a mathematical artifact of diminishing assay sensitivity. The number of secreting cells (spots) detected per well is also related to the TIL cells used. With high TIL cell numbers, we could generate 100–200 spots per well, however, resolution for counting the spots was decreased. In summary, 10–50 spots/well give us a reliable assessment of the counts, either by manual counting or computer assisted counting (data not shown). Sensitivity of our assay is similar to what described in the field when High PBMC was evaluated.

#### Calibration standard curve and linearity of dilution

Due to the lack of a standard reference material, calibration standard curves were not evaluated for quantification of cellular IFNγ response.

Linearity of dilution was evaluated using various TIL cells spiked into 4 × 10^5 ^(High PBMC) and 10^5 ^(Low PBMC) per well. IFNγ secreting cells/well at various TIL/PBMC ratios were examined. At High PBMC level, TIL1520 at 1/1250, 1/2500, 1/5000, and 1/10,000 stimulated with gp100_209 _showed dose dependent response; IFNγ secreting cells diluted from the highest number (>100 cells/well) to ~20. Good correlation was demonstrated (r^2 ^at 0.997 and 0.998 from 2 PBMC lots) using linear regression. TIL1235 at 1/625, 1/1250, 1/2500, 1/5000, 1/10,000 stimulated with MART-1 also showed dose dependent response. Correlation (r^2^) is 0.989 and 0.897 from 2 PBMC lots.

**Figure 6:**
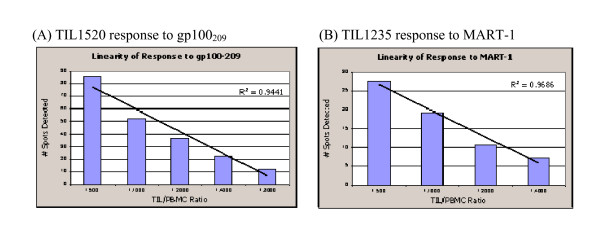
**ELISPOT linearity of dilution**. IFNγ dose response, TIL/PBMC (x-axis) vs. Secreting cells (y-axis) is shown in bar graphs and best-fit linear curve is indicated as solid line. (A) TIL1520 response to gp100_209_. (B) TIL1235 response to MART-1.

Data from Low PBMC (10^5 ^cells/well) is shown in Figure 6. Correlation (r^2^) was found to be 0.944 (gp100209) and 0.967 (MART-1).

#### Normal distribution

Eight normal PBMC samples (10^5^/well) were evaluated in normal distribution studies. Response to gp100_209_, gp100_pool_, MART-1 and HIV in all samples are below 10 IFNγ secreting cells/well. The mean (n = 8) and SD are shown in Table 8. Two samples showed low and high level of Flu response, with secreting cells at 15 and 241, respectively.

#### Determining reference ranges for assay controls

A control HLA-A2 PBMC working cell bank was established for use as an assay control. To assure plate to plate consistency, TIL1520 and TIL1235 (80 cells/well) were spiked into 10^5 ^HLA-A2 PBMC/well and were evaluated for the number of IFNγ secreting cells upon stimulation with gp100_209_, gp100_pool_, and MART-1 peptide. HIV peptide was used as a negative controls and PHA (mitogen stimulation) as positive control. Control reference ranges (Mean +/- 2 SD) were established to monitor assay performance.

### Part 4: Three validated assays demonstrated their intended use: detection of CD8+ T cell response in melanoma patients

Post-treatment PBMC obtained from three melanoma patients treated in an IRB approved melanoma vaccine protocol of the National Cancer Institute, Bethesda, MD (generously provided by Francesco Marincola) were analyzed for IFNγ response by real time RT-PCR and ELISPOT, Table 10(A) and 10(B). Response to gp100 was observed while MART-1 response was low. Tetramer analysis was not performed in our laboratory due to limited supply of the PBMC samples. Communication with Dr. Marincola confirmed that these patients demonstrated presence of gp100 tetramer positive cells (measured by Dr. F Marincola's tetramer method).

**Table 10:** Immunologic response was detected in melanoma patients after vaccination

(A) IFNγ real time RT-PCR					
**Patient**	**gp100_209_**	**gp100_209/210M_**	**gp100_pool_**	**MART-1**	**HIV**

**1**	76.9	138.1	26.2	2.5	1

**2**	3.2	3.4	6.1	0.8	1

**3**	8.5	12.5	4.5	4.5	1

HLA-A2 PBMC from three melanoma patients known to have a positive clinical response was analyzed for IFNγ response by real time RT-PCR. IFNγ response fold increase over HIV, (IFNγ _peptide_/CD8)/(IFNγ _HIV_/CD8), is shown.					

					

(B) IFNγ ELISPOT					

					

**Patient**	**gp100_209_**	**gp100_209/210M_**	**gp100_pool_**	**MART-1**	**HIV**

**1**	56	65	26	0	2

**2**	50	62	39	0	16

**3**	11	16	5	0	1

HLA-A2 PBMC from three melanoma patients known to have a positive clinical response was analyzed for IFNγ response by ELISPOT. IFNγ secreting cells (per well, average value from triplicate wells) are shown.					

					

(C) Positive MART-1 response was seen in PBMC from a melanoma patient evaluated in all three validated assays.					

					

**Method**	**ELISPOT**	**Real Time RT-PCR**	**Tetramer Assay**		

**IFNγ secreting cells**	**IFNγ copy number fold increase**	**% MART-1 tetramer positive cells**			

**Baseline** **(Pre, 11/29/00)**	0	ND	0.5		

**Post-Vaccine** **(Post-1^st ^dose, 12/20/00)**	14	5.7	1.2		

**Study Completion** **(Post 6^th ^dose, 4/18/01)**	7	4.2	2.4		

**Follow Up** **(8/15/01)**	73	57.6	4.1		

ND, not determined (insufficient cells for this analysis).

ELISPOT data is presented as the number of secreting cells after HIV background subtraction. Real time RT-PCR data is shown as copy number fold increase (over HIV) with CD8 as internal controls. Tetramer data is presented as % positive cells with negative tetramer background subtracted.

**Figure 7:**
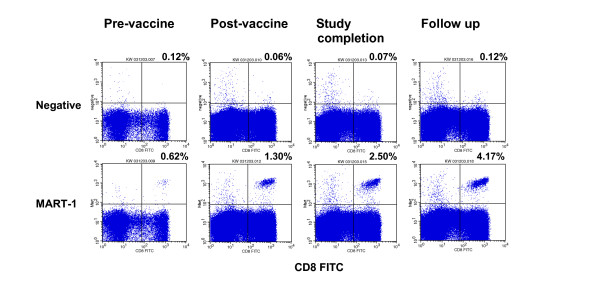
**MART-1 tetramer positive cells detected in a melanoma patient upon vaccination**. Patient's PBMC was analyzed for % MART-1 tetramer positive cells. Cells stained with negative control tetramer (upper panel) and MART-1 tetramer (lower panel) is shown. Tetramer positive cells in dot blots on gated viable lymphocytes are shown. No binding is seen among CD8 negative cell population. Percent tetramer positive cells shown are calculated based on gated viable CD8^+ ^T cells. Trend analysis demonstrated statistical significant linear response.

A representative melanoma patient who received Ad2/gp100v2 and Ad2/MART-1v2 gene therapy cancer vaccine in Genzyme Phase I/II clinical study demonstrated positive MART-1 responses measured by all three assays, Table 10(C). No gp100 specific response was observed in this patient. Compared to pre-treatment baseline response, increased MART-1 response [% MART-1 positive cells (Tetramer Assay), IFNγ fold increase (Real time RT-PCR), and IFNγ secreting cells (ELISPOT)], was observed approximately 21 days after the first dose. Increased MART-1 specific response were sustained out to study completion (after this patient received total of planned 6 doses, at ~day 140) and follow up (~day 256). Percent MART-1 tetramer positive cells are also shown in dot blots (Figure 7).

A regression analysis showed that in the tetramer assay, there is a significant linear trend between time (days) and % MART-1 positive cells with p-value of 0.0071, and the relation could be expressed as:

% MART-1 Tetramer Positive Cells = 0.0013 × days + 0.68

In the other two assays (IFNγ Real Time RT-PCR and ELISPOT), samples that were collected at the last patient's visit demonstrated a IFNγ response much higher than both the baseline response (by ELISPOT only, no RT-PCR baseline data) and earlier post vaccine time points. However, there is no statistically significant linear trend between time (days) and the IFNγ response with a p-value > 0.05 (0.3506 for Real Time RT-PCR and 0.1441 for ELISPOT).

In summary, assay performance of each assay met the validation criteria and the three validated assays demonstrated that they served their intended use.

## Discussion

The use of a wide variety of different immunoassays to assess immunological endpoints in cancer immunotherapy clinical trials has provoked recommendations that standardization and rigorous validation of these immunoassays is needed [1,11]. In response to these recommendations, we put three immunoassays, the tetramer, ELISPOT, and real time RT-PCR assays through a rigorous validation process in preparation for our cancer vaccine clinical trials. These assays met key validation criteria necessary for generating reliable clinical data. The assays were determined to be specific for each antigen, gp100 or MART-1. Assay precision for cell based functional assays met the criteria with % CV < 20% (intra day) and < 25% (inter day).

Assays were found to be sensitive with the real time RT-PCR being the most sensitive at 1 in 50,000 PBMCs. The tetramer flow cytometric method sensitivity was determined to be 1/4545–6667 (Tetramer Assay collecting 1 million events) and the ELISPOT sensitivity was at 1/10,000–20,000 (using high PBMC assay), similar to data reported by others [1]. For ELISPOT, assessment of assay sensitivity depends on number of TIL cells spiked into the number of PBMCs as the negative cell population. Due to the limited number of PBMC that could be obtained from melanoma patients, we also validated the ELISPOT assay using a low PBMC number and assay sensitivity was poor (1/2000); this is due to a mathematical calculation where responder TIL cells were spiked into a smaller PBMC population and this smaller number served as the denominator. Higher TIL cell numbers resulted in a larger number of secreting cells (100–200 cells/well), which were difficult to count due to poor resolution. We performed a TIL cell titration study and demonstrated that 10–50 cells/well provided significant resolution to achieve a reliable assessment of cell numbers.

Similarly, a larger number of total events collected for the tetramer assay will improve assay sensitivity. With limited patient PBMC samples and the need for assay throughput and cell quality (viability) during sample acquisition, we validated the tetramer assay with ~500,000 total events collected. When one million PBMC was collected, assay sensitivity was improved but samples acquired at a later time showed poor cell viability. We also evaluated the use of fixed cells after staining, and found the MFI to be much lower suggesting tetramer binding to fixed TCR was poor.

Similar differences in sensitivity between different immunoassays have been previously observed [10]. Assay sensitivity is also influenced by the T cell line (TIL cells) used to validate an immunoassay, and few groups use the same T cell lines. For example, only 33% of the cells in the TIL1520 cell line were responsive to peptide stimulation [14]. Comparisons between laboratories will likely be in closer agreement when the same cell lines are used to validate an immunoassay and same TIL cells number/PBMC number is used. As an example, the sensitivity of our ELISPOT assay was in close agreement with a previously published report where the TIL1520 were used to determine ELISPOT sensitivity [14]. A set of standard cell lines would enable a comparison of assay performance between laboratories.

While effector T cell responses can reliably be measured by each of these immunoassays, an important challenge is in determining the value that constitutes a positive response. A strong positive immunologic response measured by the MART-1 tetramer assay, such as the example shown in Figure 7, is often indisputable. Such a response profile showed a clear defined MART-1 tetramer positive CD8^+ ^T cell population that was well separated from the tetramer negative CD8^+ ^T cell population. This clearly suggests that immunization successfully enhanced the immune response. Low percentages of tetramer positive cells were seen in pre-treatment baseline sample. The binding resembles the tetramer positive cells specific for foreign antigens (Flu) in Figure 1, demonstrating breaking of tolerance to self antigen (MART-1).

On the other hand, positive responses are more likely to be detected at low percentages in the blood making it much more difficult to define a positive immunological response to a cancer vaccine. Therefore, guidelines need to be implemented on data analysis and interpretation based on assay performance characteristics such as precision and LOD. Use of proper negative controls such as the negative control tetramer, will help distinguish a positive response by setting the correct quadrant for data analysis to reduce subjectivity, especially when tetramer positive cells are not well separated from the negative population. Fold increase (>2 fold) of post-treatment response over the baseline value has been used, however, baseline values near zero value could result in an artificially high fold increase. Subtraction of the post-treatment value from the baseline value and subtraction of data from the negative control have also been used; analysis and interpretation of negative values remain challenging.

Interestingly, MART-1 tetramer positive CD8+ T cells were detected among both healthy volunteers and in melanoma patients who received cancer vaccines. Among healthy volunteers, MART-1 positive cells showed low MFI (median) probably reflecting low affinity/avidity (MFI is not shown). In patients, however, MART-1 positive cells had high MFI (Figure 7). Function of these MART-1 positive CD8^+ ^T cells were reported that the cells in healthy volunteers may be of naïve phenotype, which lacks effector function (presence of CTL precursors); however, in cancer patients, these cells have the memory phenotype [15-17]. Their effector function was demonstrated *in vitro *upon MART-1 peptide stimulation (in the presence of APC such as dendritic cells) using methods such as cytokine production (IL-2, GM-CSF, IFNγ) and CTL activity.

Correlation of MART-1 specific CD8^+ ^T cells in peripheral blood with the presence of CTL cells at the tumor site and clinical response *in vivo *is still not fully established [15,17-20]. A majority of the peptide reactive CD8^+ ^cells may not be tumor reactive due to various mechanisms such as down modulation of HLA class I on tumor cell surface and presence of regulatory T cells and TGFβ, etc. Sorting of the tetramer positive cells for generation of CTL *in vitro *has been used as adoptive transfer (cell based therapy) in melanoma patients [21,22]. Correlation of immunologic response to clinical response (tumor regression) still needs to be established [15,17,20]. MART-1 specific CD8^+ ^T cell response in one patient, as an example, was detected by all three validated clinical assays, HLA-A2 MART-1 tetramer assay, IFNγ real time RT-PCR and ELISPOT (Figure 7 and Table 10), demonstrating the assay utility in monitoring patient T cell response. Correlation to clinical response, however, was not demonstrated. A better understanding of the immune response seen in peripheral blood vs. the response at the tumor site will help us more fully understand the mechanisms of cancer vaccine and its potency.

The use of validated methods for clinical patient monitoring is important. When following patient longitudinal responses over time, our understanding of assay performance will assist us in implementing procedures that reduce assay variability and the use of QC samples will allow us to monitor assay long term performance; making data generated from these validated methods more meaningful. While the validation of these three T cell assays was challenging, the experience we obtained during validation studies and conducting patient screening will assist us and others in the field to validate similar assays for assessment of patient T cell responses to not only to cancer vaccines but to other therapeutic proteins as part of immunogenicity and safety analysis.

## Conclusion

In this manuscript, we reported data from validation studies to characterize three T cell assays, HLA-A2 tetramer Flow cytometric method, IFNγ real time RT-PCR, and IFNγ ELISPOT for detection of gp100 or MART-1 specific CD8^+ ^T cell response.

Although challenging, our results showed that T cell functional assays can be validated to support clinical longitudinal sample testing to monitor patient T cell response to cancer vaccines. All three assays demonstrated their intended use for detection of cancer vaccine specific T cell response (Figure 7 and Table 10). Use of validated assays in clinical patient monitoring minimized assay reproducibility problems and allowed better interpretation of clinical data.

## Abbreviations

Ad2, Adenovirus 2. CD4 or CD8, cluster of differentiate 4 or 8, helper T (CD4) and cytotoxic T (CD8) cells.  DC, Dendritic cell. ELISPOT, enzyme linked immunospot assay. Gp100, melanoma tumor antigen. HIV, human immunodeficiency virus. HLA, human leukocyte antigen; HLA-A2, HLA allele A*0201. MART-1, melanoma tumor antigen. PBMC, peripheral blood mononuclear cell  PCR, polymerase chain reaction.

## Competing interests

All authors are Genzyme employees except KD and KS who were formal Genzyme employees.  We have received salary from Genzyme Corporation.

## Authors' contributions

YX, wrote the manuscript, led the tetramer assay study, and analyzed data for ELISPOT and RT-PCR studies. VT, reviewed the manuscript and led the ELISPOT study. CS, led the RT-PCR study. KD, acquired and analyzed tetramer data. LA, acquired and analyzed RT-PCR data. KS, acquired and analyzed ELISPOT data. MAP, supervised pre-validation studies for tetramer, RT-PCR, and ELISPOT and assisted writing the manuscript. SMR, supervised all validation studies for tetramer, RT-PCR, and ELISPOT and gave  final approval of the version to be published.
